# Draft Genome Sequence of Enterococcus faecium Strain J19, Isolated from Cabbage

**DOI:** 10.1128/genomeA.00213-18

**Published:** 2018-04-05

**Authors:** Diana I. Ayala, Peter W. Cook, David L. Campos, Jorge G. Franco, Mindy M. Brashears, Henk den Bakker, Kendra K. Nightingale

**Affiliations:** a International Center for Food Industry Excellence, Department of Animal and Food Sciences, Texas Tech University, Lubbock, Texas, USA

## Abstract

Herein, we report the draft genome sequence of a newly discovered probiotic strain, Enterococcus faecium J19, which was isolated from cabbage. Strain J19 has shown antagonistic effects against the human foodborne pathogen Listeria monocytogenes in coculture and in different food matrices.

## GENOME ANNOUNCEMENT

Probiotics are live microorganisms that benefit a host by improving the intestinal microbial balance (1). The most frequently used probiotic strains are lactic acid-producing bacteria from the genera Lactobacillus, Enterococcus, Streptococcus, and Bifidobacterium (2). Species of the genus Enterococcus are ubiquitous in nature and have been shown to naturally inhabit the gastrointestinal tracts of mammals, plant material, and a wide range of foods (3, 4). Enterococcus species produce a variety of compounds that inhibit spoilage and pathogenic microorganisms, including enzymes, aromatic compounds, and bacteriocins (3, 5, 6), which has led to increased interest in the use of Enterococcus strains in food preservation and food safety and as probiotics in animal feed to replace the subtherapeutic use of antibiotics (7).

Enterococcus faecium is one of the main species of the genus Enterococcus, and it has been commonly isolated from food and clinical samples (7). The bacteriocinogenic activity of E. faecium against closely related species and other Gram-positive bacteria, such as species of the genus Listeria, has been previously reported (3). E. faecium strain J19 was isolated from cabbage; experiments in our laboratory have shown its efficacy in reducing Listeria monocytogenes in cantaloupe by 1.06 log_10_ CFU/ml after 1 day of dipping and by 0.5 log_10_ CFU/ml on stainless steel after 4 h of application (our unpublished data). E. faecium J19 has also been effective at reducing L. monocytogenes by 3.3, 2.4, and 2.0 log_10_ CFU/ml in queso fresco, cheddar, and Babybel cheeses, respectively (our unpublished data). Strain J19 is intended to be used as a food safety application for dipping and/or spraying cheeses to reduce L. monocytogenes cell counts. Specific features of strain J19 are highlighted in this report.

Enterococcus faecium J19 was cultivated in MRS broth, and genomic DNA was isolated using the Invitrogen Purelink DNA extraction kit (Thermo Fisher Scientific, Waltham, MA). DNA was used for library preparation with a Nextera XT kit v2.0 (San Diego, CA). DNA libraries were paired-end sequenced using a 2 × 250-base pair (bp) v2 kit on an Illumina MiSeq platform. Raw reads were filtered using Trimmomatic v0.33 (8) and *de novo* assembled into 95 scaffolds with an estimated size of 2,777,899 bp and an average G+C content of 40.3% using SPAdes v3.5 (9). The final *N*_50_ and *N*_90_ values of the genome were 102,571 and 21,379 bp, respectively, with a genome coverage of 110×. The J19 draft genome sequence was annotated using the NCBI Prokaryotic Genome Annotation Pipeline (10), and 2,721 coding sequences, 61 tRNAs, and 3 rRNAs were identified. Five putative bacteriocins (three enterocins, one enterolysin, and one lactacin) were identified by the BActeriocin GEnome mining tooL 3 (BAGEL3) (11), two potential prophages were identified by the PHAge Search Tool Enhanced Release (PHASTER) Web server (12), and one contig containing a potential Enterococcus plasmid was identified by PlasmidFinder (13). Additionally, one gene for potential resistance to macrolides, lincosamides, and streptogramin B was identified when the sequence was compared against the ResFinder database (14), and 15 potential virulence factors were identified when it was compared against the Virulence Factors Database (VFDB) (15).

### Accession number(s).

This whole-genome shotgun sequencing project has been deposited in DDBJ/ENA/GenBank under the accession no. PHUR00000000. The version described in this paper is the first version, PHUR01000000.
